# Association between genetic risk variants and glucose intolerance during pregnancy in north Indian women

**DOI:** 10.1186/s12920-018-0380-8

**Published:** 2018-08-08

**Authors:** Geeti P. Arora, Peter Almgren, Charlotte Brøns, Richa G. Thaman, Allan A. Vaag, Leif Groop, Rashmi B. Prasad

**Affiliations:** 1 Deep Hospital, Ludhiana, Punjab India; 2 Department of Clinical Sciences, Clinical Research Centre, Lund University, Malmö, Sweden; 3 Department of Endocrinology (Diabetes and Metabolism), Rigshospitalet, Copenhagen, Denmark; 4 Finnish Institute of Molecular Medicine (FIMM), Helsinki University, Helsinki, Finland; 5 Cardiovascular and Metabolic Disease (CVMD) Translational Medicine Unit, Early Clinical Development, IMED Biotech Unit, AstraZeneca, Gothenburg, Sweden

**Keywords:** Genetics, Risk variant, Gestational diabetes mellitus, Single nucleotide polymorphism, Diagnostic criteria, Insulin resistance, Insulin secretion, Type 2 diabetes mellitus

## Abstract

**Background:**

Gestational diabetes (GDM) is a more common problem in India than in many other parts of the world but it is not known whether this is due to unique environmental factors or a unique genetic background. To address this question we examined whether the same genetic variants associated with GDM and Type 2 Diabetes (T2D) in Caucasians also were associated with GDM in North Indian women.

**Methods:**

Five thousand one hundred pregnant women of gestational age 24–28 weeks from Punjab were studied by a 75 g oral glucose tolerance test (OGTT). GDM was diagnosed by both WHO1999 and 2013 criteria. 79 single nucleotide polymorphisms (SNPs) previously associated with T2D and glycemic traits (12 of them also with GDM) and 6 SNPs from previous T2D associations based on Indian population (some also with European) were genotyped on a Sequenom platform or using Taqman assays in DNA from 4018 women.

**Results:**

In support of previous findings in Caucasian GDM, SNPs at *KCJN11* and *GRB14* loci were nominally associated with GDM1999 risk in Indian women (both *p* = 0.02). Notably, T2D risk alleles of the variant rs1552224 near *CENTD2,* rs11708067 in *ADCY5* and rs11605924 in *CRY2* genes associated with protection from GDM regardless of criteria applied (*p* < 0.025). SNPs rs7607980 near *COBLL1* (*p* = 0.0001), rs13389219 near *GRB14* (*p* = 0.026) and rs10423928 in the *GIPR* gene (*p* = 0.012) as well as the genetic risk score (GRS) for these previously shown insulin resistance loci here associated with insulin resistance defined by HOMA2-IR and showed a trend towards GDM. GRS comprised of 3 insulin secretion loci here associated with insulin secretion but not GDM.

**Conclusions:**

GDM in women from Punjab in Northern India shows a genetic component, seemingly driven by insulin resistance and secretion and partly shared with GDM in other parts of the world. Most previous T2D loci discovered in European studies did not associate with GDM in North India, indicative of different genetic etiology or alternately, differences in the linkage disequilibrium (LD) structure between populations in which the associated SNPs were identified and Northern Indian women. Interestingly some T2D risk variants were in fact indicative of being protective for GDM in these Indian women.

**Electronic supplementary material:**

The online version of this article (10.1186/s12920-018-0380-8) contains supplementary material, which is available to authorized users.

## Background

Gestational Diabetes Mellitus (GDM) has been officially defined as “carbohydrate intolerance” of variable severity with onset or first recognition during pregnancy [1–3] irrespective of treatment and whether or not the condition persists after pregnancy. GDM represents almost 90% of all pregnancies complicated by diabetes [4]. The prevalence of GDM is rapidly increasing, ranging from 2 to 14% depending upon diagnostic criteria [5, 6]. In a study of South Indian women, GDM prevalence varied between 12 and 21% [7] while another study of North Indian women reported a prevalence of 10% using WHO criteria [8]. The hallmark of GDM is increased insulin resistance accompanied by decreased compensatory insulin secretory response. Type 2 diabetes (T2D) is also caused by increased insulin resistance and decreased insulin secretion to compensate for the former. Thus, both T2D and GDM share the same pathophysiology which is influenced by similar risk factors like high body mass index (BMI), history of abnormal glucose intolerance, family history of diabetes, age, and ethnicity [9–11].

A family history of both T2D and GDM is known to increase GDM risk, indicative of a common genetic component underlying both T2D and GDM [12, 13]. Till date, more than 120 T2D risk loci have been confirmed to be associated with T2D [14]. A large proportion of them have also shown association with GDM. T2D risk variants at the *MTNR1B, FTO, TLE1, G6PC2, GCKR, TCF7L2, ADCY5, CDKAL1, TCF2, HNF1B, PPARG, KCNJ11, SLC30A8* loci have previously been associated with GDM in European populations [15–18] whereas variants in the *CDKAL1, CDKN2A/2B, MTNR1B* and *KCNQ1* loci were associated with GDM in Korean women [19, 20].

Some genetic variants are more unique to Indian T2D patients e.g. the *SGCG* (rs9552911) and *TMEM163* (rs998451) variants [21–25]. However, genetic studies of GDM in India are scarce. The SNPs rs7754840 and rs7756992 in the *CDKAL1* gene were associated with GDM in South Indian women [26], while variants in the *HMG20A* (rs7178572) and *HNF4A* (rs4812829) genes were associated with both GDM and T2D [27]. The aim of the present study was to investigate whether a panel of known variants previously associated with GDM and T2D in Indian and European populations are associated with GDM in Punjabi women.

## Methods

### Study population and phenotyping

Five thousand one hundred pregnant women were recruited by applying a multistage random screening in the State of Punjab in North India for GDM. Pregnant women at gestational week 24–28 were randomly selected and recruited [8, 28]. This was part of a WDF supported project titled “Gestational diabetes in Punjab” with the goal to create and implement sustainable awareness, education, screening, intervention and treatment capacities of diabetes in pregnancy (GDM) within the public and private health care system, as well as in the general population in Punjab. The team included a chief research coordinator, an assistant coordinator, doctors, nurses, lab technicians from all selected sites both in private hospitals and public healthcare system. Approval for screening was obtained from DRME, Chandigarh, India. The recruitment sites included Recruitment sites:, Deep Hospital, Model Town, Ludhiana as the epicenter, Shri Rama Charitable Hospital, Ludhiana, Chawla Hospital, Ludhiana, Iqbal Hospital, Ludhiana, Government Medical Colleges and Hospital, Patiala, Amritsar and Faridkot, PHC Verka, Amritsar, Health Centre Bhadsoan, Patiala, Health Centre Faridkot. The project was approved by Independent ethics committee, Ludhiana in 2009. The ethics committee is registered with Office of Drugs Controller General (India) Directorate General of Health Services with Registration no. ECR/525/Inst/PB/2014.

Information was obtained on age, BMI, family history of diabetes, diet, habitat (urban or rural), education and religion. All information material and written consent forms were provided in 3 languages (Hindi, Punjabi & English) and duly signed by the participants. The study protocol was approved by local Ethical Committees. Glucose was measured in venous plasma samples at fasting and at 2 h after a 75 g glucose challenge using glucometers (Accucheck-Roche Diagnostics). Fasting insulin concentrations were determined with ELISA (Diametra, Milan, Italy; intra- and inter-assay variation of < 5.0 and < 10.0%, respectively)**.** The homeostatic model assessment (HOMA2) was used to quantify insulin resistance (HOMA2-IR) and beta-cell function (HOMA2-B) from fasting insulin and glucose values using the HOMA2 calculator v2.2.3 (http://https://www.dtu.ox.ac.uk/homacalculator/) [29]. GDM was diagnosed according to the WHO1999 (FPG ≥7.0 mmol/l and/or 2-h glucose ≥7.8 mmol/l) and the adapted WHO2013 (FPG ≥5.1 and/or 2-h glucose ≥8.5 mmol/l) criteria (ref). The clinical characteristics of subjects are shown in Table 1.

**Table 1:** Study population characteristics

				GDM1999			Controls			GDM2013			Controls		
	N	Mean	±SD	N	Mean	±SD	N	Mean	±SD	N	Mean	±SD	N	Mean	±SD
Age (years)	4018	21.41	3.40	346	21.11	3.59	3672	21.44	3.38	1386	21.68	3.5	2632	21.27	3.34
BMI	4018	24.11	4.34	346	24.28	4.71	3672	24.09	4.30	1386	24.36	4.48	2632	23.97	4.25
Fasting plasma glucose (mmol/l)	4018	4.81	0.76	346	5.53	1.32	3672	4.74	0.65	1386	5.51	0.69	2632	4.44	0.49
Plasma insulin (pmol)	4018	54.25	61.86	346	46.73	42.24	3672	54.96	63.35	1386	52.74	54.44	2632	55.05	65.43
2 h glucose (venous, mmol/l)	4018	6.20	1.37	346	9.15	1.83	3672	5.93	0.92	1386	6.85	1.70	2632	5.86	1.00
homa2_b with steady state glucose and insulin values	3680	104.02	55.71	346	78.01	37.56	3672	106.36	56.49	1386	77.37	38.02	2632	117.92	58.36
homa2_ir with steady state glucose and insulin values	3680	0.97	0.74	346	0.96	0.73	3672	0.97	0.74	1386	1.02	0.79	2632	0.95	0.71

### Genotyping

DNA was extracted from frozen and stored buffy coats using (QIAGEN Autopure LS kits. Six SNPs previously associated with GDM or T2D in India [21, 22, 26, 27, 30] (Additional file 2: Table S1) and 79 SNPs previously associated with T2D in Europe and elsewhere from GWAS studies up to 2012 (some of these also with GDM risk from candidate gene studies in GDM populations) were genotyped in the present study (Additional file 2: Table S1) [14] on a Sequenom Mass ARRAY Platform (Sequenom San Diego, CA, USA) PLEX using MALDI-TOF mass spectrometer [31] or Taqman allelic discrimination assays using an ABI Prism 7900 sequence detection system (Applied Biosystems, Foster City, CA, USA). Genotyping was performed at the Lund University Diabetes Centre, Sweden after obtaining permission from ICMR (dated 21 october 2010 and Office of Drugs Controller General (India)(dated 14/12/2010).

Replication genotyping of 6% of the samples showed > 98% concordance. rs6467136, and rs7202877 had a Hardy-Weinberg equilibrium (HWE) *p*-value of < 0.001 in unaffected women based on WHO1999 criteria and < 0.05 in unaffected women based on WHO2013 criteria and were hence removed from the analysis.

### Statistical analyses

Association of selected SNPs with risk of GDM was assessed by logistic regression analysis adjusted for maternal age and BMI and results presented as ORs with 95% confidence intervals (CI). We also tested for associations with fasting and 2-h glucose values as well as with fasting insulin and HOMA2-B and HOMA2-IR (Additional file 2: Table S1) using linear regression analysis with maternal age and BMI as covariates. Individuals with missing data were excluded. Data were logarithmically transformed before analysis. The power to detect association with GDM2013 including 1386 GDM women and 2632 controls at *p* < 0.0006 (0.05/79) (after Bonferroni correction) for a SNP allele frequency of 0.3 and effect size 1.3 was 0.97, which decreased to 0.64 for effect size 1.2 under an additive model. For GDM1999, with 346 GDM and 3672 controls, the corresponding figures were 0.39 and 0.12 respectively. For association with quantitative glucose traits, power to detect association was 1 at alpha 0.05 for and allele frequency of 0.3 [32, 33]. A *p*-value of ≤0.05 was considered statistically significant on account of the current analyses being replication of previously published associations.

Genetic risk scores for insulin secretion (HOMA-2B) and insulin resistance (HOMA-2IR) were calculated using SNPs previously associated with insulin secretion and insulin resistance. SNPs were assessed for linkage disequilibrium (LD) and for those in high LD (r^2^), only one representative SNP was retained. Individual scores were calculated based on number of risk alleles weighed by their effect sizes reported in previous GWAS studies and logistic regression was performed against normalized measures of insulin secretion and insulin resistance.

All calculations were implemented in STATA, plink 1.09 and SPSS v22.0.

## Results

Among the 4018 genotyped women, applying the WHO2013 criteria resulted in a total of 1386 women with GDM (34.5%) whereas the number was reduced to 346 (8.6%) when WHO1999 criteria were used. Notably, only 283 (7.0%) women were diagnosed using both GDM 2013 and GDM 1999 criteria (Additional file 1: Figure S1) [34]. This is concordant with our previously published reports on the larger subset of the same population comprising 5100 women [28]. HOMA2-B was lower in GDM women defined by both criteria compared to pregnant normal glucose tolerant women (PNGT). HOMA2-IR was also higher in women with GDM2013 who thereby were more insulin resistant than PNGT (Table 1).

### SNPs previously associated with GDM/T2D in India

None of the 8 SNPs previously associated with GDM or T2D in Indian populations was here associated with GDM (Table 2). However, analysis for association with GDM1999 or GDM 2013 against controls who did not satisfy either criterion revealed the nominal association of rs7756992 in *CDKAL1* while rs689 in *INS* showed a trend towards association with GDM2013 (Table 3).

**Table 2:** Association of previously reported GDM and T2D loci from Indian population based studies with risk of GDM according to both criteria

Genotype	EA	Chr	Gene/nearest gene	Location	OR_WHO1999	lower CI	upper CI	p_who1999	OR_WHO2013	lower CI	upper CI	p_who2013	n
rs998451	A	2	*TMEM163*	intron	0.987	0.795	1.224	0.902	0.959	0.843	1.09	0.518	3882
rs1799999	A	7	*PPP1R3A*	missense	0.862	0.728	1.02	0.083	0.997	0.905	1.098	0.953	3890
rs689	A	11	*INS*	5’UTR	1.077	0.879	1.319	0.474	1.033	0.914	1.167	0.603	3903
rs9552911	A	13	*SGCG*	intron	1.057	0.83	1.347	0.653	1.017	0.875	1.183	0.824	3890
rs4812829	A	20	*HNF4A*	intron	1.04	0.871	1.24	0.667	0.988	0.89	1.096	0.814	3801
rs7178572	G	15	*HMG20A*	intron	0.988	0.832	1.173	0.891	1.017	0.921	1.122	0.743	3541
rs7756992	G	6	*CDKAL 1*	intron	0.91	0.75	1.1	0.34	0.97	0.87	1.08	0.64	3686
rs7754840	C	6	*CDKAL1*	intron	0.87	0.72	1.06	0.17	0.96	0.86	1.07	0.51	3721

*EA* effect allele, *OR_WHO1999* odds ratio based on WHO1999 criteria, *OR_WHO2013* Odds ratio based on WHO2013 criteria, *CI* confidence interval

**Table 3:** Association of previously reported GDM loci with risk of GDM according to both criteria

SNP	EA	Chr	Gene/nearest gene	Location	WHO 1999				WHO 2013				n
					OR	CI(lower)	CI(upper)	*p*-value	OR	CI(lower)	CI(upper)	*p*-value	
rs9939609	A	16	*FTO*	intron	1.04	0.86	1.26	0.67	0.98	0.88	1.10	0.83	3120
rs2796441	G	9	*TLE 1*	intergenic	0.99	0.84	1.16	0.92	1.07	0.97	1.17	0.15	3905
rs560887	C	2	*G6PC2/ABCB11*	intron	1.18	0.92	1.52	0.19	1.11	0.96	1.28	0.13	3910
rs11708067	A	3	*ADCY5*	intron	0.98	0.81	1.18	0.86	0.88	0.79	0.99	**0.037**	3877
rs1111875	C	10	*HHEX*	intergenic	0.90	0.77	1.06	0.22	1.05	0.96	1.16	0.24	3901
rs10811661	T	9	*CDKN2A/2B*	intergenic	0.99	0.77	1.26	0.93	1.08	0.94	1.25	0.23	3890
rs4402960	T	3	*IGF2BP2*	intron	1.02	0.87	1.20	0.77	0.95	0.86	1.04	0.29	3750
rs13266634	C	8	*SLC30A8*	coding-missense	0.96	0.79	1.17	0.75	0.97	0.87	1.08	0.61	3898
rs7903146	T	10	*TCF7L2*	Intronic/promoter	1.13	0.95	1.35	0.14	1.01	0.916	1.12	0.76	3543
rs10830963	G	11	*MTNR1B*	intron	0.89	0.75	1.05	0.20	0.98	0.89	1.08	0.69	3714
rs1801282	C	3	*PPARG*	Coding-missense	0.86	0.89	1.12	0.22	0.99	0.93	1.08	0.21	3652
rs10010131	G	4	*WFS1*	intron	1.13	0.95	1.36	0.16	0.99	0.90	1.10	0.99	3843
rs5219	T	11	*KCNJ11*	coding-missense	1.21	1.03	1.42	**0.019**	1.00	0.90	1.10	0.99	3595

*EA* effect allele, *OR_WHO1999* odds ratio based on WHO1999 criteria, *OR_WHO2013* Odds ratio based on WHO2013 criteria, *CI* confidence interval

significant *p* values where *p* < 0.05 are indicated in bold

### Previously reported GDM risk loci

Out of 12 selected previously reported GDM risk loci, the T allele of the missense SNP rs5219 in the *KCNJ11* gene was nominally associated with GDM1999 (*p* = 0.019) (Table 4). Contrary to previous reports, the risk allele A of SNP rs11708067 in the *ADCY5* gene showed reduced risk for GDM defined by 2013 (*p* = 0.037) (Table 4) but not by 1999 criteria. The SNP rs2796441 in the *TLE1* gene was associated with decreased insulin secretion (*p* = 0.013) (Additional file 2: Table S2). The rs13266634 at *SLC30A8* locus associated with GDM1999 while SNPs rs5219 in *KCNJ11* and rs11708067 in *ADCY5* associated with GDM2013 nominally when controls satisfying neither GDM diagnosis criteria were considered (Table 3).

**Table 4:** Association of previously reported T2D loci with risk of GDM according to both criteria

SNP	EA	Chr	Gene/nearest gene	Location	WHO 1999				WHO 2013				n
					OR	CI(lower)	CI(upper)	*p*-value	OR	CI(lower)	CI(upper)	*p*-value	
rs2296172	G	1	*MACF1*	coding-missense	0.92	0.71	1.20	0.56	1.04	0.89	1.21	0.58	3847
rs340874	C	1	*PROX1*	intergenic	0.94	0.80	1.11	0.52	0.96	0.87	1.06	0.47	3709
rs7578597	T	2	*THADA*	coding-missense	0.90	0.72	1.12	0.37	0.92	0.80	1.06	0.27	3710
rs243088	T	2	*BCL 11A*	intergenic	1.10	0.94	1.29	0.22	1.07	0.97	1.18	0.15	3717
rs7593730	T	2	*RBMS1/ITGB6*	intronic	1.01	0.84	1.22	0.83	0.99	0.88	1.11	0.93	3906
rs7607980	C	2	*COBLL1*	coding-missense	0.95	0.73	1.24	0.75	0.95	0.81	1.11	0.52	3885
rs13389219	C	2	*GRB14*	intergenic	1.25	1.03	1.52	**0.022**	1.11	0.99	1.23	0.058	3829
rs7578326	A	2	*KIAA1486/IRS1*	intron of uncharacterized LOC646736	0.97	0.80	1.18	0.78	0.98	0.87	1.10	0.79	3600
rs2943641	C	2	*IRS1*	intergenic	0.92	0.76	1.12	0.43	0.97	0.87	1.09	0.67	3643
rs4675095	A	2	*IRS1*	intron	1.11	0.87	1.42	0.39	1.04	0.90	1.19	0.58	3817
rs831571	C	3	*PSMD6*	intergenic	1.02	0.84	1.25	0.77	0.93	0.83	1.05	0.26	3726
rs4607103	C	3	*ADAMTS9-AS2*	intron	1.14	0.98	1.33	0.08	1.00	0.91	1.09	0.97	3884
rs11920090	T	3	*SLC2A2*	intron	1.19	0.93	1.51	0.16	1.16	1.01	1.33	**0.03**	3606
rs6815464	C	4	*MAEA*	intron	1.04	0.83	1.30	0.71	1.03	0.90	1.18	0.64	3722
rs459193	G	5	*ANKRD55*	intergenic	0.99	0.84	1.16	0.90	1.07	0.97	1.18	0.16	3884
rs4457053	G	5	*ZBED3*	intron of ZBED3-AS1	1.05	0.86	1.29	0.57	0.95	0.84	1.07	0.45	3579
rs9470794	C	6	*ZFAND3*	intron	1.07	0.85	1.35	0.51	1.05	0.91	1.21	0.48	3608
rs17168486	T	7	*DGKB*	intergenic	0.99	0.83	1.17	0.92	0.97	0.88	1.07	0.62	3855
rs2191349	T	7	*DGKB/TMEM195*	intergenic	1.04	0.88	1.22	0.62	1.00	0.91	1.10	0.95	3903
rs864745	T	7	*JAZF1*	intron	0.98	0.83	1.16	0.87	1.02	0.92	1.13	0.68	3876
rs4607517	A	7	*GCK*	intergenic	1.04	0.82	1.32	0.70	1.01	0.88	1.16	0.86	3903
rs17133918	C	7	*GRB10*	intron	1.03	0.87	1.23	0.67	0.97	0.88	1.08	0.65	3907
rs933360	A	7	*GRB10*	intron	1.03	0.87	1.22	0.70	1.03	0.93	1.14	0.54	3905
rs6943153	C	7	*GRB10*	intron	0.86	0.73	1.03	0.11	0.95	0.86	1.05	0.36	3602
rs516946	C	8	*ANK1*	intron	1.01	0.82	1.23	0.91	1.09	0.97	1.23	0.13	3922
rs896854	T	8	*TP53INP1*	intron	0.97	0.83	1.14	0.75	0.97	0.88	1.06	0.57	3903
rs7034200	A	9	*GLIS3*	intron	0.98	0.83	1.15	0.84	1.03	0.93	1.13	0.52	3868
rs13292136	C	9	*TLE4 (CHCHD9)*	intergenic	0.94	0.75	1.18	0.62	0.98	0.86	1.12	0.79	3706
rs12571751	A	10	*ZMIZ1*	intron	0.86	0.73	1.01	0.07	0.96	0.87	1.06	0.49	3601
rs553668	A	10	*ADRA2A*	UTR-3	1.17	0.99	1.39	0.06	1.07	0.97	1.19	0.15	3666
rs10885122	G	10	*ADRA2A*	intergenic	1.03	0.84	1.27	0.75	1.05	0.93	1.18	0.42	3683
rs163184	G	11	*KCNQ1*	intron	0.90	0.76	1.07	0.23	1.00	0.90	1.10	0.98	3713
rs2237895	C	11	*KCNQ1*	intron	0.96	0.81	1.13	0.66	1.01	0.92	1.11	0.79	3682
rs11605924	A	11	*CRY2*	intron	0.84	0.72	0.97	**0.025**	1.00	0.92	1.10	0.85	3909
rs7944584	A	11	*MADD*	intron	0.91	0.74	1.13	0.41	1.09	0.96	1.23	0.15	3553
rs174550	T	11	*FADS1*	intron	0.94	0.76	1.17	0.62	0.96	0.85	1.09	0.56	3908
rs1552224	A	11	*CENTD2*	intergenic	0.92	0.75	1.13	0.45	0.81	0.72	0.92	**0.001**	3911
rs11063069	G	12	*CCND2*	intergenic	0.99	0.80	1.23	0.98	1.04	0.91	1.19	0.52	3671
rs10842994	C	12	*KLHDC5*	intergenic	1.13	0.89	1.44	0.28	0.97	0.84	1.11	0.67	3906
rs1153188	A	12	*DCD*	intergenic	1.15	0.93	1.42	0.19	1.01	0.89	1.14	0.82	3912
rs1531343	C	12	*HMGA2*	intron of pseudogene	0.83	0.67	1.03	0.09	0.90	0.80	1.02	0.10	3915
rs7961581	C	12	*TSPAN8,LGR5*	intergenic	0.91	0.77	1.08	0.31	1.02	0.92	1.13	0.61	3703
rs7957197	T	12	*OASL/TCF1/HNF1A*	intron of QASL	0.87	0.65	1.17	0.37	1.00	0.83	1.21	0.96	3924
rs17271305	G	15	*VPS13C*	intron	1.02	0.86	1.20	0.81	0.92	0.83	1.02	0.15	3825
rs11071657	A	15	*FAM148B*	intergenic	1.03	0.87	1.22	0.72	0.92	0.83	1.02	0.13	3897
rs7177055	A	15	*HMG20A*	intergenic	1.00	0.85	1.17	0.99	0.98	0.89	1.08	0.74	3907
rs35767	G	12	*IGF1*	nearGene-5	0.88	0.91	1.10	0.19	0.93	0.94	1.06	0.21	3910
rs11634397	G	15	*ZFAND6*	intergenic	0.89	0.76	1.04	0.16	0.96	0.87	1.06	0.47	3910
rs8042680	A	15	*PRC1*	intron	0.89	0.76	1.04	0.16	0.99	0.90	1.10	0.95	3887
rs8090011	G	18	*LAMA1*	intron	0.95	0.81	1.11	0.57	0.93	0.84	1.02	0.13	3911
rs10401969	C	19	*SUGP1*	intron	0.96	0.72	1.27	0.79	0.86	0.72	1.01	**0.07**	3605
rs8108269	G	19	*GIPR*	intergenic	1.02	0.85	1.23	0.77	1.07	0.96	1.19	0.16	3508
rs10423928	A	19	*GIPR*	intron	0.85	0.67	1.08	0.20	1.06	0.93	1.20	0.37	3911
rs6017317	G	20	*FITM2-R3HDML-HNF4A*	intergenic	0.96	0.81	1.13	0.64	0.98	0.89	1.08	0.72	3758
rs5945326	A	X	*DUSP9*	intergenic	0.95	0.81	1.12	0.58	1.01	0.92	1.12	0.74	3589

*EA* effect allele, *OR_WHO1999* odds ratio based on WHO1999 criteria, *OR_WHO2013* Odds ratio based on WHO2013 criteria, *CI* confidence interval

significant *p* values where *p* < 0.05 are indicated in bold

### Previously reported T2D loci

The risk allele C of SNP rs13389219 in the *GRB14* gene was associated with GDM1999 (*p* = 0.022) (Table 5) but not with GDM2013 (*p* = 0.058) (Table 5). The T2D risk allele T of SNP rs11920090 in the intron of the *SLC2A2* gene was associated with GDM2013 (*p* = 0.030) (Table 5).

**Table 5:** Sensitivity analysis for association of selected risk variants with GDM risk

SNP	EA	Chr	Gene/nearest gene	Location	WHO 1999					WHO 2013				n
					OR	CI(lower)	CI(upper)	*p*-value	n	OR	CI(lower)	CI(upper)	*p*-value	
rs13266634^a^	T	8	*SLC30A8*	coding-missense	1.24	1.01	1.53	**0.037**	2834	1.049	0.91	1.21	0.50	3837
rs11605924	A	11	*CRY2*	intron	0.84	0.71	0.99	**0.038**	2833	1.005	0.91	1.10	0.91	3848
rs35767	T	12	*IGF1*	nearGene-5	1.26	1.00	1.60	0.054	2837	1.15	0.98	1.33	0.07	3848
rs5219^a^	T	11	*KCNJ11*	coding -missense	1.18	1.00	1.40	0.059	2605	1.00	0.91	1.11	0.91	3539
rs11708067^a^	G	3	*ADCY5*	intron	1.11	0.86	1.44	0.42	2810	1.25	1.09	1.45	**0.002**	3816
rs689^a^	A	11	*INS*	Promoter/intron	0.91	0.64	1.29	0.60	2835	0.81	0.65	1.00	0.054	3842
rs8108269	G	19	*GIPR*	intergenic	1.14	0.94	1.36	0.17	2568	1.12	0.99	1.25	0.059	3449
rs7756992^a^	G	6	*CDKAL1*	intron	0.96	0.76	1.19	0.69	2670	2.80	1.00	7.87	**0.049**	3626

^a^indicates loci previously associated with GDM / T2D in India or GDM in studies based on the European population

Logistic regression was performed on GDM cases diagnosed according to WHO1999 and WHO2013 criteria against controls who had no GDM diagnosis using either criteria

significant *p* values where *p* < 0.05 are indicated in bold

Surprisingly, the T2D risk allele A of SNP rs11605924 in the *CRY2* gene was associated with reduced risk of GDM1999 (*p* = 0.025) (Table 5). The same variant associated with GDM1999 in a sensitivity analysis when controls meeting neither GDM diagnosis criteria were considered (Table 3). In support of this, the same allele was also associated with lower 2-h glucose levels (*p* = 0.038) (Additional file 2: Table S3).

The risk allele A of SNP rs1552224 in the *CENTD2* locus was associated with decreased risk of GDM2013 (*p* = 0.001) (Table 5).

### Association with insulin secretion and insulin resistance

Twelve SNPs previously associated with insulin secretion were here tested for association with HOMA2-B. The T2D risk allele A of rs11071657 at the *FAM148B* locus was nominally associated with increased insulin secretion (*p* = 0.044) (Table 6). A GRS comprising of 3 previously reported insulin secretion loci with the lowest *p*-values for insulin secretion in the present study associated with insulin secretion in the present study (*p* = 0.008, beta = 0.25, SE = 0.098). GRS for insulin secretion did not associate with either GDM2013 (*p* = 0.15, beta = − 0.06, SE = 0.045) or GDM1999 (*p* = 0.73, beta = − 0.009, SE = 0.026).

**Table 6:** Association of selected loci with insulin secretion (HOMA2-B)

SNP	EA	Chr	Gene/nearest gene	Location	Beta	SE	*p*-value	N
rs340874	C	1	*PROX1*	intergenic	0.009	0.011	0.388	3395
rs560887	C	2	*G6PC2/ABCB11*	intron	−0.004	0.016	0.818	3578
rs11708067	A	3	*ADCY5*	intron	−0.024	0.012	0.053	3556
rs11920090	T	3	*SLC2A2*	intron	−0.014	0.015	0.361	3301
rs4607517	A	7	*GCK*	intergenic	0.007	0.012	0.571	3372
rs2191349	T	7	*DGKB/TMEM195*	intergenic	−0.008	0.011	0.480	3575
rs7034200	A	9	*GLIS3*	intron	0.002	0.016	0.922	3576
rs10885122	G	10	*ADRA2A*	intergenic	−0.006	0.010	0.546	3545
rs7944584	A	11	*MADD*	intron	−0.021	0.013	0.116	3372
rs7903146	T	10	*TCF7L2*	Intronic/promoter	0.003	0.011	0.798	3240
rs10830963	G	11	*MTNR1B*	intron	−0.007	0.011	0.473	3398
rs174550	T	11	*FADS1*	intron	0.011	0.014	0.435	3248
rs7756992	G	6	*CDKAL1*	intron	0.011	0.014	0.446	3576
rs11071657	A	15	*FAM148B*	intergenic	−0.023	0.011	**0.044**	3568

significant *p* values where *p* < 0.05 are indicated in bold

Of 6 SNPs previously associated with measures of insulin resistance, 3 SNPs here associated with HOMA2-IR. The C allele of rs7607980 in the *COBLL1* gene was associated with decreased HOMA2-IR (*p* = 0.0001). The C allele of rs13389219 near *GRB14* (*p* = 0.026) and A allele of rs10423928 in the intron of the *GIPR* gene (*p* = 0.012) showed worse insulin resistance (increased HOMA2-IR; Table 7). Genetic risk scores (GRS) calculated based on the 3 SNPs associated with insulin resistance showed an increase of insulin resistance by 0.07 (SE = 0.145, *p* = 0.006) per allele. GRS for insulin resistance showed a trend towards GDM2013 (*p* = 0.065, beta = 0.076, SE = 0.04) but not GDM1999 (*p* = 0.14, beta = 0.023, SE = 0.025).

**Table 7:** Association with HOMA-IR selected loci: insulin resistance SNPs

SNP	EA	Chr	Gene/nearest gene	Location	Beta	SE	*p*-value	N
rs2943641	C	2	*IRS1*	intergenic	−0.001	0.014	0.923	3337
rs4675095	A	2	*IRS1*	intron	−0.028	0.017	0.102	3500
rs4607517	A	7	*GCK*	intergenic	0.018	0.018	0.299	3576
rs7607980	C	2	*COBLL1*	coding-missense	0.070	0.019	**0.0001**	3557
rs13389219	C	2	*GRB14*	intergenic	0.029	0.013	**0.026**	3518
rs10423928	A	19	*GIPR*	intron	0.041	0.016	**0.012**	3585

significant *p* values where *p* < 0.05 are indicated in bold

## Discussion

In this large study, we investigated the genetic basis of gestational diabetes mellitus in Punjabi Indian women [15, 16, 19, 27].

Surprisingly, the genetic variants in the *HMG20A* and *HNF4A* genes which previously have been associated with risk of T2D and GDM in South India [27] were not associated with GDM or T2D in Punjabi pregnant women. This could be due to differences in allele frequencies between the North and South Indian populations, which are ethnically quite distinctive populations [35]. The Punjabi Indian population belongs to the “Ancestral North Indians” group and shares genetic similarities with populations from Middle East, Central Asia and to some degree Europe whereas the South Indian population genetically belongs to the distinct “Ancestral South Indian” group [35]. Notably the *CDKAL1* variant associated with GDM only when a sensitivity analysis was performed using controls that had no GDM diagnosis using either GDM1999 or GDM2013 criteria, thus replicating a previous association.

Neither did we observe associations with loci associated with GDM elsewhere including variants in the *CDKAL1* and *MTNR1B* loci, which have been reported to be associated with GDM in South Korea [19]. A sensitivity analysis using controls that had no GDM diagnosis using either criterion revealed the nominal association of variants in *SLC30A8*, *KCNJ11* and *ADCY5*. These largely negative findings could be attributed to population-based differences. Previous studies have indicated differences in anthropometry between Indian and European populations, with the former manifesting a “thin-fat” phenotype [36]. Subsequently, it is possible that since most T2D loci were identified in European ancestry cohorts, the negative findings could reflect differences in tagging SNPs due to differences haplotypes between populations. On the other hand, the underlying etiology of GDM could also be different genetically. While the study population is the largest GDM study till date, this might lack sufficient power to detect genome-wide significance levels of association with an unstable phenotype. The effect sizes of previously reported T2D loci were low, generally under odds ratios of 1.2, therefore the study was not sufficiently powered to demonstrate association of SNPs with such low effect sizes. Alternately, considering the lack of consensus for GDM diagnosis criteria worldwide, it is plausible that this could be due to different thresholds that might apply for the Indian population.

Notably, T2D risk variants in the *CRY2* (WHO1999), *CENTD2* (WHO2013) and the *ADCY5* (WHO2013) genes were here protective for GDM. *CRY2* encodes for the cryptochrome protein involved in the regulation of the circadian clock. Risk allele carriers of the rs11708067 SNP in *ADCY5* has previously been shown to reduce *ADCY5* expression in pancreatic beta cells and important for coupling glucose to insulin secretion in human islets [37]. It has been previously shown that T2D risk alleles show extreme directional differentiation across various populations, with T2D risk alleles decreasing in frequency along human migration into East Asia [38]. Such flip-flops of risk alleles may be explained by population differences, possibly due to genetics or environment. Alternately, such “flip-flop” associations have also been attributed to multi-locus effects as shown from theoretical modeling studies demonstrating that the direction of allelic effect may flip when tested allele is inversely correlated with another risk allele at another locus, or positively correlated with a protective allele at another locus [39].

A HWE threshold of < 0.001 in unaffected individuals based in either criteria was set as a cut-off; SNPs showing significant deviations from HWE should be interpreted with caution, since these could be indicative of population substructures, inbreeding or selection. The current study only comprises genotyping data from candidate SNPs which do not provide sufficient coverage of the genome to detail population stratification or inbreeding. HWE could also be indicative of actual association. A serious problem in the study of the genetics of GDM is the implementation of different criteria, since some women could be classified as controls based on different criteria. For SNP rs5219 in *KCNJ11* (HWE *p* = 0.004, WHO1999; HWE *p* = 0.01, WHO2013) and rs11605924 in *CRY2* (HWE *p* = 0.007 WHO1999 and HWE *p* = 0.06, WHO2013), HWE values were nominally significant for the same criteria where an association was observed; these findings need to be replicated in independent cohorts.

Of 6 loci previously associated with insulin resistance, here 3 also showed an association with HOMA2-IR and a trend towards significance for GDM2013 but not GDM1999 including SNPs rs7607980 in the *COBLL1* gene [40], rs13389219 near *GRB14* and rs10423928 in the *GIPR* gene indicating that some of the genetic basis seem to be driven by previously reported insulin resistance loci. Similarly, a GRS with the 3 variants with the lowest *p*-values for insulin secretion associated with insulin secretion but not GDM2013 or GDM1999.

Taken together, the results demonstrate that GDM in women from Punjab in Northern India shows a genetic component, partially shared with GDM in other parts of the world, and seems to be driven by both insulin resistance and secretion. However, the direction of the effect can differ; some T2D risk variants were indicative of being protective for GDM in these Indian women.

## Conclusions

GDM in women from Punjab in Northern India shows a genetic component shared with T2D. This genetic basis is seemingly driven by a complex interplay between insulin secretion and sensitivity during pregnancy and is at least partly shared with GDM in other parts of the world. Interestingly some of the T2D risk variants in *ADCY5* and *CRY2* were protective against GDM. Most of the previous T2D loci discovered in European studies did not associate with GDM in North India. Interestingly some T2D risk variants were in fact indicative of being protective for GDM in these Indian women. This could be attributed to different genetic etiology or differences in the LD structure between populations in which the associated SNPs were identified and Northern Indian women. GWAS or whole genome sequencing will be interesting to further unravel the genetic basis of GDM in India.

## Additional file

Additional file 1: **Figure S1.** Number of GDM women according to WHO2013 and WHO1999 criteria. (PDF 81 kb)

Additional file 2: **Table S1.** T2D M associated SNPs selected from previously published GWAS studies upto 2012 and GDM associated loci from previous candidate and GWAS studies. (*) indicate SNPs previously associated with GDM or T2D in India. **Table S2. ** Association of previously reported GDM loci with glycemic traits. **Table S3.** Association of GDM loci identified in the current study with glycemic traits. (XLSX 30 kb)

## Abbreviations

GDM: Gestational diabetes mellitus
GRS: Genetic risk score
GWAS: Genome wide association study
HOMA2: Homeostatic model assessment
HOMA2-B: Homeostatic model assessment for insulin secretion
HOMA2-IR: Homeostatic model assessment for insulin resistance
LD: Linkage disequilibrium
OGTT: Oral glucose tolerance test
SNP: Single nucleotide polymorphism
T2D: Type 2 diabetes
