# Supplementary figures and images for: Association between genetic risk variants and glucose intolerance during pregnancy in north Indian women
Source: BMC Med Genomics. 2018 Aug 8;11:64. doi: 10.1186/s12920-018-0380-8 (PMC6083526; doi:10.1186/s12920-018-0380-8)

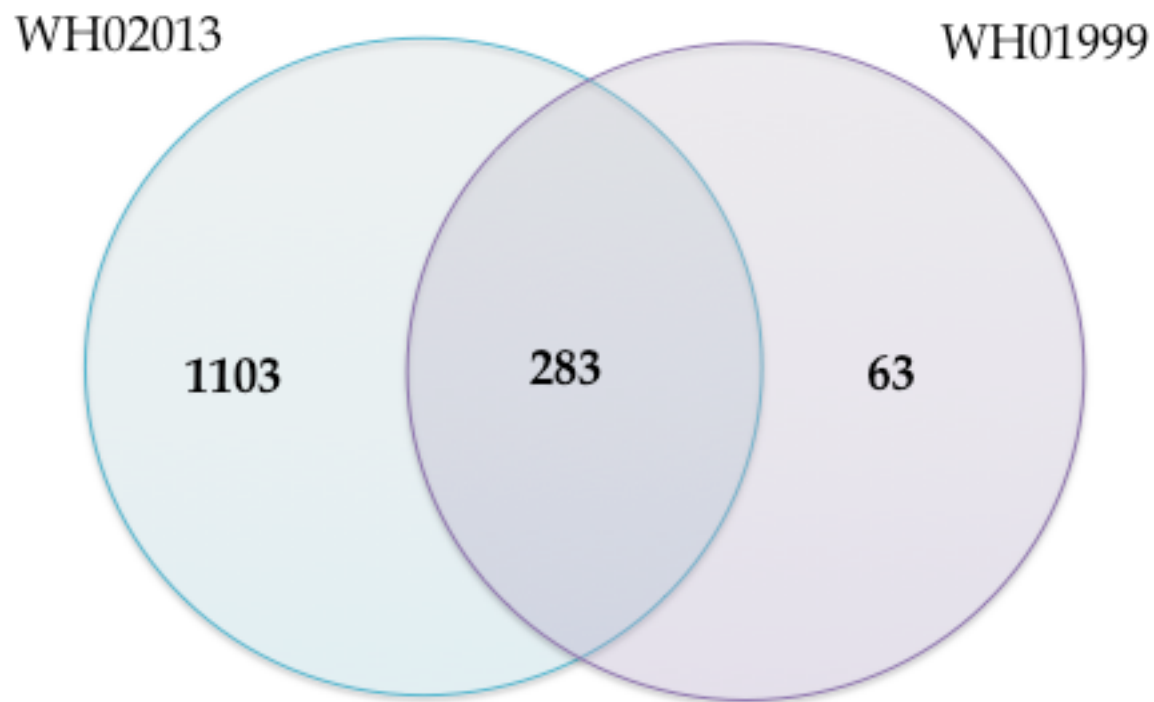

**Supplementary Figure 1. Number of GDM women according to WHO2013 and WHO1999 criteria**

Supplement: Supplementary file 1 — Figure S1. Number of GDM women according to WHO2013 and WHO1999 criteria. (PDF 81 kb) [file 12920_2018_380_MOESM1_ESM.pdf]
